# Measuring patient outcome using data capture by mobile app

**DOI:** 10.1186/1745-6215-16-S1-P27

**Published:** 2015-05-29

**Authors:** Carol Fawkes, Robert Froud, Dawn Carnes

**Affiliations:** 1 Barts and The London School of Medicine and Dentistry, London, E1 2AB, UK; 2 University of Warwick, Coventry, CV4 7AL, UK; 3 Norges Helsehøyskole Campus Kristiania, 0152 Oslo, Norway

## 

The use of Patient Reported Outcome Measures (PROMs) to measure effectiveness of care, and supporting patient management is being advocated increasingly in clinical and research settings. Current patient data capture involves completion of paper questionnaires which is costly and environmentally challenging. New innovations are required to balance the challenges of introducing data capture directly from patients while considering budgets, access to Information Technology, and the capability to use technological devices.

The development of content for a mobile and web app for capturing PROM data has been informed by two qualitative studies, and a systematic review. The qualitative studies involved interviews and focus groups with patients and clinicians (osteopaths) concerning their views on using PROMs in clinical practice, and a selection of specific PROMs. The systematic review compared the measurement properties of three PROMs (the Roland Morris Disability Questionnaire, the Oswestry Disability Index, and the Bournemouth Questionnaire).

Patients (N=18) have been enthusiastic about using PROMs in practice welcoming the opportunity to provide feedback, and undaunted by use of technology. It was shown to be important to include PROMs with numerical scales, and text descriptions of symptoms. Clinicians (N=30) also recognised the value of PROMs and the importance of outcome data being collected independently of their clinics. However, there were some concerns. Patients wanted clarity concerning the use of data, and to whom it would be accessible. In addition patients were concerned about the potential disruption to the consultation process. Clinicians were concerned about fitting the collection of PROM data into the consultation process, and how this could affect the relationship with patients.

The findings of these three studies have informed the development of a mobile and web app. Both apps are currently being piloted in private and training clinics for osteopaths.

